# Evaluation of Change in Radiographic Fractal Dimension around Dental Implants Placed with Low-Speed Drilling and Standard Drilling Protocols

**DOI:** 10.3390/jcm12062244

**Published:** 2023-03-14

**Authors:** Sofía Soler-Alcaraz, Yolanda Guerrero-Sánchez, Mario Pérez-Sayáns, Juan Carlos Bernabeu-Mira, David Peñarrocha-Oltra, Fabio Camacho-Alonso

**Affiliations:** 1 Department of Oral Surgery, University of Murcia, 30100 Murcia, Spain; 2 Department of Human Anatomy and Psicobiology, University of Murcia, 30100 Murcia, Spain; 3 Health Research Institute Foundation of Santiago (FIDIS) (ORALRES Group) Oral Medicine, Oral Surgery and Implantology Unit, Faculty of Medicine and Dentistry, University of Santiago de Compostela (MedOralRes Group), 15782 Santiago de Compostela, Spain; 4 Oral Surgery Unit, Department of Stomatology, Faculty of Medicine and Dentistry, University of Valencia, Gascó Oliag 1, 46010 Valencia, Spain

**Keywords:** fractal dimension, bone trabeculation, bone density, dental implantology, cone beam computed tomography, orthopantomography

## Abstract

Osseointegration is a process that depends on a multitude of factors, including the type of drilling, whether biological or conventional. Objective: Establish box-counting dimension values for radiological images in patients with implants placed with both drilling methods. Material and method: The sample included 129 implants corresponding to 50 patients. A double-blind study of data collection was carried out with the subsequent analysis of the fractal dimension as a comparative value of the state of the trabecular architecture. Results: We found no significant differences (*p* ≥ 0.05) between the two study groups comparing both drilling techniques. The values for the conventional drilling technique are 0.24 ± 0.07 and for biological drilling: 0.19 ± 0.11 with a *p*-value of 0.767. Conclusions: The drilling technique does not influence the success of the procedure and the osseointegration process.

## 1. Introduction

The alveolar bone itself has a high turnover rate throughout life, being dependent on multiple factors. This evolution over the years translates into a formation of new bone when the masticatory function increases and a decrease when it is smaller [1]. At the same time, external factors such as smoking or systemic internal factors such as diabetes can lead to changes in the alveolar bone. In the same way, after an implant surgery, a bone remodeling known as osseointegration takes place, which will constitute a direct relationship between bone and implant, lacking periodontal tissue. The phases of this process are:

- Osteoconduction: phase in which a bed of connective tissue and blood coagulation appears around the implant, which will mature over time into granulation tissue.
- Modeling: osteoclasts begin to appear, gradually reabsorbing the avascular bone. At the same time, the connective tissue rich in vessels will mature forming an osteoid that will form the reticular bone.
- Remodeling: the reticular bone is replaced by lamellar bone and bone marrow, with the intervention of osteoclasts. By the eighth week, all the original bone portions will have been replaced by new bone [2].

Oral implantology is one of the dental sciences that has had the greatest impact and evolution for the oral rehabilitation of patients, given its high possibilities and success in those cases where the possibilities of rehabilitation were complicated due to the decrease in bone and the absence of many teeth [3]. Much of the success achieved in these treatments is based on the high planning and predictability that image analysis offers us over these types of radiological tests. At a quantitative level, all the measurements made on the maxillary and mandibular bone allow us to improve treatment decisions and future prediction of success.

The fractal dimension (FD) is one of the methods used to quantitatively measure the complex geometric units (fractals) that constitute an image. This method is used in radiodiagnosis to identify the bone trabecular pattern linked to bone quality. It has been shown that by measuring the FD in periapical radiographs, minimal peri-implant changes in the trabecular structure can be detected [4,5], and the same happens in studies that use this method in panoramic or CBCT radiology [6].

In the field of dentistry, it has been applied for more than 40 years, but at the level of implants it began to be applied after the studies carried out on the bone trabecular structure [7,8,9,10].

During this type of surgery, various parameters are taken into account to achieve the subsequent osseointegration of the implants: type of bone in relation to density by Housfield measurements, distance between bone crests, height of the dental alveolus,... In the preparation of the bed during bone drilling, the fact of conducting the surgery towards a process that is as “atraumatic” as possible so that the success of the treatment is more predictable.

During the osteotomy, heat is always generated on contact with the bone; however, there are thermal limits of 47–50 Celsius degrees from which the peri-implant bone will tend to necrosis. On the other hand, the milling speed causes controversy, finding values that range between 50 and 2500 rpm [11]. There is evidence that highlights milling at low revolutions (50 rpm–100 rpm), as it allows greater control in direction and depth. And in turn, a lower increase in bone temperature facilitates the collection of autologous bone during the intervention for later use. Using this technique, the bone obtained preserves a large part of its cellular vitality and can be used in peri-implant defects or gaps [12].

Given that bone density is closely related to the applied drilling technique, through this study we intend to evaluate the bone trabecular structure before and after implant placement through the FD value, differentiating two study groups subjected to conventional drilling and at low speed during the surgical phase. We start from the hypothesis that there should be no differences between both groups.

## 2. Materials and Methods

### 2.1. Design of the Study

We carried out a retrospective and double-blind longitudinal study from October 2020 to January 2022, previously approved by the bioethics commission of the University of Murcia ID: 3203/2021, obtaining the sample of patients belonging to the Master of Oral Surgery and Implantology Hospital Morales Meseguer. All procedures were carried out with due understanding and written consent of the subjects in accordance with the Declaration of Helsinki.

### 2.2. Type of Sample, Inclusion and Exclusion Criteria

We start from a sample of 50 patients (n = 50), with 129 implants analyzed on the corresponding images (CBCT and orthopantomographies). The patients belong to two different groups depending on the type of drilling performed in the placement of the implants. The examiners do not know which patients belong to one or another group, therefore considering the double-blind study. The types of milling meet the following characteristics:

1. Low-speed drilling without irrigation: 50 rpm.
2. Conventional drilling with irrigation: 800 rpm.

All patients meet the established inclusion and exclusion criteria:

1. Patients of legal age and not pregnant.
2. Patients who do not present pathologies that affect the bone.
3. Insufficient quality images.
4. Not having pre and post implants images.

### 2.3. Obtaining Images and Processing

All panoramic radiographs were performed using the same digital panoramic radiography system (Vatech, Madrid, Spain).

The CBCT images obtained were performed by the same Planmeca^®^ team, Planmeca ProMax 3-D Max (Planmeca Oy, Helsinki, Finland), duly calibrated. The radiographs were taken with the patients in the prone position, adjusting their head position using the device’s laser guidance system. The emission parameters of the beam of rays were kV = 96, Ma = 8, exposure time of 12 s (11.94 s) and an image size of 501 × 501 × 466 voxels (each voxel being equivalent to 200 µm).

The evaluation software used was the Romexis 2.5.1.R program (Planmeca Oy, Helsinki, Finland), which allowed viewing the image in a multiple window where the axial, coronal and sagittal planes can be viewed at 0.2 mm intervals in addition to a 3D vision.

**Figure 1:**
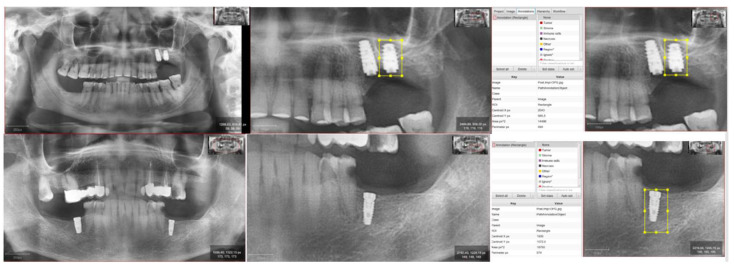
Selection of ROI on image of the maxilla and mandible, with description of the size.

We establish a region of interest (ROI) of a differentiated study in the maxilla and mandible on orthopantomographic projections with different sizes detailed in the figure (Figure 1). We work on images obtained in two phases:

- Diagnostic or pre-implantation phase, CBCT obtained before the surgical procedure.
- Post-implantation phase, CBCT obtained after implant placement and with prosthetic load.

The implants belong to different commercial houses: BTI^®^ (Biotechnology Institute S.L., Vitoria, Spain), Galimplant^®^ (Nueva Galimplant, S.L.U., Lugo, Spain) and Biomet 3i^®^ (Biomet 3i Dental Ibérica S.L, Barcelona, Spain).

For the processing and analysis of the images we work with two different software: qupath (Quantitative Pathology & Bioimage Analysis. Center for Cancer Research & Cell Biology at Queen’s University Belfast) and ImageJ (National Institutes of Health).

**Figure 2:**
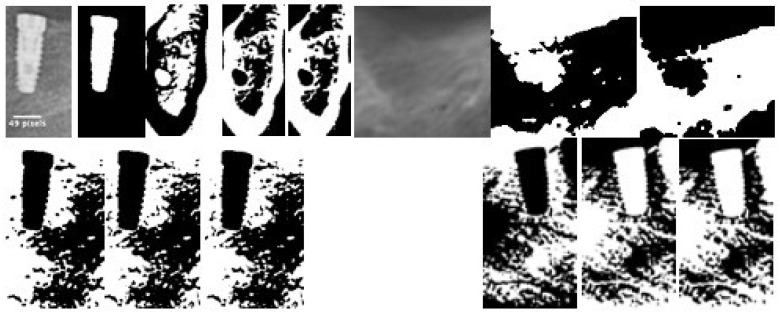
Image processing sequence to apply the White and Rudolph method.

Radiological processing is performed according to the White and Rudolph method for radiological images [13]. As can be seen in Figure 2, starting from the original image, we create a mask that allows us to cut out the area corresponding to the implant so that it does not interfere with the FD calculation. Once cropped, as we work with 8-bit images, we perform thresholding, erosion and two dilations, having previously transformed it to binary, to calculate the FD value.

### 2.4. Fractal Dimension Analysis

The fractal dimension is a mathematical invariant that allows to find patterns of self-similarity in structures. This value is mainly computed using the so-called box-counting dimension, but the results generated in many case are not informative enough due to the non-accuracy of the output value. In the present work, we shall compute the fractal dimension using a novel algorithm stated by Y. Guerrero et al. at [14]. The idea is to use images of high resolution where the new tool, which does not compute boxes like the classical box counting but use the preimages of the intersection of a recovering curve at the image treat.

### 2.5. Statistic Analysis

Data were analyzed by using SPSS 20.0 statistics program (SPSS^®^ Inc., Chicago, IL, USA). A descriptive study was made of each variable. The Kolmogorov-Smirnov normality test and Levene variance homogeneity test were applied, and the data showed a normal distribution, and were analyzed using parametric tests. The associations between the different qualitative variables were studied using Pearson’s chi squared test. The associations between different quantitative variables were studied using the student *t*-test for two related samples. Statistical significance was accepted for *p* ≤ 0.05.

## 3. Results

The sample turned out to be homogeneous for the demographic characteristics studied, as shown in Table 1, both in age, sex and in the tobacco item. The sample consists of 50 patients with a mean age of 54.64 ± 12.52 (30–72) and a gender distribution of 26 men (52%) and 24 women (48%). No significant differences were found when making the comparisons (Table 1).

Regarding the characteristics directly related to the implants, they are described in Table 2, we take into consideration: type of implant, placement area (maxillary or mandibular), anatomical section (anterior or posterior), length and dental position of the implant.

**Figure 3:**
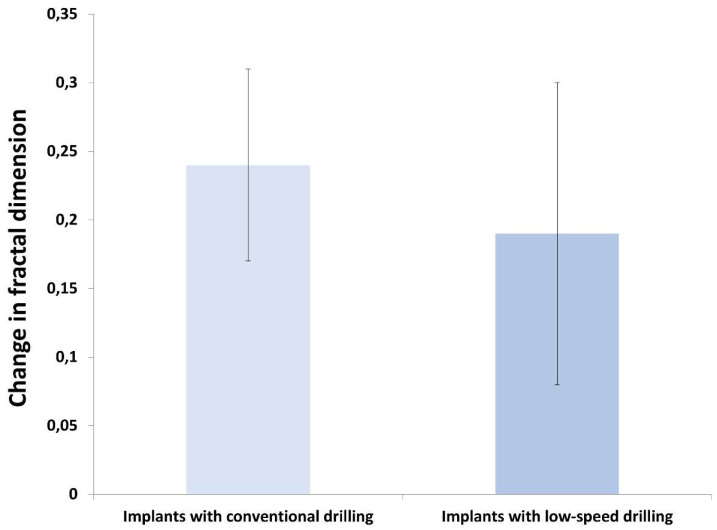
Comparison of change in fractal dimension (pre and postsurgical) between study groups (* *p* ≤ 0.050).

Regarding the study of the fractal dimension, we found significant differences in the values between the pre-implant and post-implant images in both groups, (see Table 3). There are no significant differences between the FD values of the two study groups as shown in Figure 3.

## 4. Discussion

- The assessment of the stability of the implants and the changes produced during the osseointegration process through the radiographic study have been analyzed by numerous authors. To the point of becoming a crucial requirement to assess the changes produced around the implant, both at the bone and soft tissue level [15,16].
- Regarding the different radiological possibilities that technology offers us, most authors have traditionally used it to analyze the bone density of the bone area that concerns us in surgery [17,18].
- However, other authors have used the study of the fractal dimension to assess the state of the bone trabeculate due to its structural [19,20]. In the present paper we stated as a null hypothesis that there were no differences between the two groups subjected to conventional drilling and at low speed during the surgical phase. As a conclusion of our study, we cannot reject this null hypothesis.
- The trabecular architecture complies with the mathematical definition of a fractal, as a complex structure that meets a series of criteria, including self-similarity. We could affirm that we analyze both the maxillary and mandibular bones from the orientation that we want to use (be it distal, mesial, vestibular, or lingual) and we always find partitions and spaces resembling a network.
- Based on this criterion, our study states the analysis of the patient’s bone individually, establishing an initial value before implant placement and then compare the different values obtained in the radiological examinations established by the protocol. It shall allow us to establish an optimal measure of assessment of small architectural changes at the trabecular level. Changes mediated by phases of bone resorption and apposition that take place around the implant and that end in its own osseointegration [21,22,23].

Other authors maintain that the success of the implant in its osseointegration process and its adequate evolution depends on a set of circumstances, such as the correct preparation and drilling of the area, without giving the weight of surgical success to the type of drilling performed [24].

On the other hand, various authors focus on the techniques used for implant placement, highlighting that they condition the results obtained when evaluating the success rate and the osseointegration processes [25,26,27]. Different factors can influence:

1. Temperature during drilling.
2. Revolutions in drilling.

Several in vitro studies have demonstrated that temperature during low-speed drilling without irrigation was always lower than the critical threshold temperature exposure of 47 °C for one minute [28,29,30,31,32,33].

Clinically, the low-speed technique has demonstrated a high implant success rate similar to that the one obtained with standard drilling. Histopathologically, several experimental studies showed the same osteointregation process for both techniques without statistically significant differences. The present study showed the same results as other analytical test such as fractal dimensional study [34,35,36,37,38].

All these conditions lead to differentiating between conventional drilling and biological drilling, seeking which could be the most effective for the success of the implant osseointegration process.

- Bernabeu-Mira et al. [27] carried out a systematic review on both types of reaming, concluding that most of the studies carried out obtained the same results as ours, no significant differences were found regarding osseointegration and success rate between both types of reaming. Regardless of the technique used to assess drilling, almost all of them are analyzed both pathologically and radiologically.
- Sukanya Mishra et al. [39], in their review work, conclude that the fractal dimension may be a value that, together with conventional methods, helps to assess the stability of the implant. They analyze the results obtained in the different studies that use this method and correlate it with those obtained with conventional methods. We consider that with the limitations that the method presents, it can also at the radiographic level, being a minimally invasive technique, provide a lot of information in the times of the osseointegration process.
- In recent times, this value is becoming very important in the field of dentistry, being applied in various processes, not only in implantology [40,41]. Even in other fields such as dermatology, ophthalmology… [42,43]. Which, in part, supports its application to the bone itself in surgical processes, since the results of most of the studies conclude that the fractal dimension is a value that presents advantages and seems to give conclusive results when it comes to quantifying bone density.
- Kulczyk et al. [15] study the stability of the implant through the fractal dimension, concluding that it cannot be ensured that this measurement by itself is a sufficient value to determine the stability of the implant. Likewise, various authors establish that there is no consensus on the relationship between the fractal dimension and the complexity of the bone, but they do admit that certain changes in bone density occur [44,45].
- Most of these articles where no correlation is found are quite old, so the radiographic technique could have some influence, since the quality of images obtained today is much higher, especially if we are talking about CBCT.

Other authors have found that the fractal dimension values vary after the intervention to return to the original values that the patient had before the intervention over a period, as occurs in our study [46].

The fractal dimension values must be analyzed individually and considering the bone type because is not the same from a morphological point of view the maxillary and the jaw bone. According to various authors, this fact could condition the FD value due to the trabecular complexity of the maxillary bone. Although, we can establish a value interval where the non-pathological bone is recognized [47] as we do in the present work.

## 5. Conclusions

Considering the limitations of the study, we can conclude that the fact that there are no significant differences between the two groups indicates that the type of drilling does not affect the quality of the bone or the osseointegration process. The fractal dimension is a good indicator to assess the important changes produced in the bone trabeculae, so that it offers the clinician a worthy support to evaluate the changes produced in the osseointegration process. More studies are needed to seed clarity and consensus on this issue.

## Figures and Tables

**Table 1:** Homogeneity of the study groups in terms of the demographic characteristics and habits (Student *t*-test and Pearson χ^2^).

Characteristics	Patients with Conventional Drilling (n = 25)	Patients with Low-Speed Drilling (n = 25)	*p*-Value
Age: mean ± SD *	56.52 ± 11.95	52.76 ± 13.03	0.293
Sex: n (%)			1.000
Male	13 (52)	13 (52)	
Female	12 (48)	12 (48)	
Smoking behaviour: n (%)			0.269
Non-smoker	19 (76)	22 (88)	
smoker	6 (24)	3 (12)	

* SD = standard deviation.

**Table 2:** Implant distribution.

Characteristics	Total (n = 129) n (%)	Implants with Conventional Drilling (n = 65) n (%)	Implants with Low-Speed Drilling (n = 64) n (%)
Dental implant type			
BTI^®^	60 (46.52)	37 (56.92)	23 (35.93)
Galimplant^®^	33 (25.57)	0 (0)	33 (51.56)
Biomet3i^®^	36 (27.91)	28 (43.08)	8 (12.51)
Maxilla/Mandible			
Maxilla	60 (46.52)	27 (41.54)	33 (51.56)
Mandible	69 (53.48)	38 (58.46)	31 (48.44)
Length			
8 mm	3 (2.32)	0 (0)	3 (4.68)
8.5 mm	10 (7.75)	7 (10.76)	3 (4.68)
10 mm	73 (56.58)	37 (56.92)	36 (56.25)
11.5 mm	27 (20.93)	15 (23.07)	12 (18.75)
12 mm	12 (9.31)	2 (3.10)	10 (15.64)
13 mm	4 (3.11)	4 (6.15)	0 (0)
Diameter			
3.25 mm	3 (2.32)	3 (4.54)	0 (0)
3.50 mm	11 (8.52)	0 (0)	11 (17.18)
3.75 mm	23 (17.84)	10 (15.38)	13 (20.31)
4.00 mm	80 (62.01)	45 (69.23)	35 (54.68)
4.50 mm	12 (9.31)	7 (10.76)	5 (7.83)
Site			
1.14	(3.11)	1 (1.53)	3 (4.68)
1.22	(1.55)	1 (1.53)	1 (1.56)
1.35	(3.87)	3 (4.54)	2 (3.12)
1.46	(4.65)	2 (3.10)	4 (6.25)
1.55	(3.87)	1 (1.53)	4 (6.25)
1.68	(6.21)	5 (8.07)	3 (4.68)
2.13	(2.32)	0 (0)	3 (4.68)
2.24	(3.11)	2 (3.10)	2 (3.12)
2.36	(4.65)	3 (4.54)	3 (4.68)
2.46	(4.65)	4 (6.15)	2 (3.12)
2.57	(5.46)	3 (4.54)	4 (6.25)
2.65	(3.87)	2 (3.10)	3 (4.68)
2.72	(1.55)	1 (1.53)	1 (1.56)
3.11	(0.77)	0 (0)	1 (1.56)
3.23	(2.32)	2 (3.10)	1 (1.56)
3.32	(1.55)	1 (1.53)	1 (1.56)
3.42	(1.55)	1 (1.53)	1 (1.56)
3.55	(3.87)	1 (1.53)	4 (6.25)
3.616	(12.41)	9 (13.84)	7 (10.93)
3.74	(3.11)	4 (6.15)	0 (0)
4.12	(1.55)	1 (1.53)	1 (1.56)
4.21	(0.77)	0 (0)	1 (1.56)
4.32	(1.55)	1 (1.53)	1 (1.56)
4.41	(0.77)	0 (0)	1 (1.56)
4.55	(3.87)	3 (4.54)	2 (3.12)
4.617	(13.17)	11 (16.92)	6 (9.47)
4.75	(3.87)	3 (4.54)	2 (3.12)

**Table 3:** Presurgical and postsurgical fractal dimension in study groups (Student *t*-test).

Fractal Dimension			
Groups	Presurgical Mean ± SD *	Postsurgical Mean ± SD	*p*-Value
Implants with conventional drilling (n = 65)	1.68 ± 0.06	1.71 ± 0.05	<0.001
Implants with low-speed drilling (n = 64)	1.67 ± 0.08	1.69 ± 0.07	<0.001

* SD = standard deviation.

## Data Availability

All data used in the present paper are contained in the manuscript.
